# Analysis of ASMs and male infertility using the FDA adverse event reporting system (FAERS)

**DOI:** 10.3389/fpubh.2025.1738546

**Published:** 2026-01-26

**Authors:** Kunyang An, Jingyu Xu, Yujia Xi, Xinfang Cao, Jingqi Wang, Xiaoqin Hu, Xuezhi Liang

**Affiliations:** 1 Department of Urology Surgery, First Hospital of Shanxi Medical University, Taiyuan, Shanxi, China; 2 The First Clinical Medical College, Shanxi Medical University, Taiyuan, Shanxi, China; 3 Department of Epidemiology, School of Public Health, Shanxi Medical University, Taiyuan, Shanxi, China; 4 Research Centre of Environmental Pollution and Major Chronic Diseases Epidemiology, Shanxi Medical University, Taiyuan, Shanxi, China; 5 Department of Urology, Second Hospital of Shanxi Medical University, Taiyuan, Shanxi, China; 6 Male Reproductive Medicine Center, Shanxi Medical University, Taiyuan, Shanxi, China; 7 Shanxi Medical University, Taiyuan, Shanxi, China

**Keywords:** disproportionality analysis, drug safety, FAERS, male infertility, pharmacovigilance

## Abstract

**Introduction:**

Antiseizure medications (ASMs) are the cornerstone of epilepsy treatment, but their potential reproductive toxicity may impact male fertility. Globally, approximately 15% of couples are affected by infertility, with male factors accounting for 50%. This study aimed to analyze the association between ASMs and male infertility using the FDA Adverse Event Reporting System (FAERS).

**Methods:**

Data from January 1, 2004, to September 30, 2024, were extracted from FAERS. A hybrid signal detection framework combining non-Bayesian (Reporting Odds Ratio, ROR) and Bayesian (Bayesian Confidence Propagation Neural Network, BCPNN) methods was employed to evaluate the reporting frequency and risk of male infertility adverse events for different ASMs.

**Results:**

Among 81,618 deduplicated case reports involving specified ASMs, 60 were related to male infertility. Disproportionality analysis revealed that carbamazepine (ROR = 8.73; IC = 3.10) and valproic acid (ROR = 6.82; IC = 2.74) posed the highest risks. Oxcarbazepine, lamotrigine, and levetiracetam also showed positive signals. Phenytoin sodium, topiramate, and clonazepam showed no significant risk. Regarding overall ASM-related reports, the majority originated from the United States and involved patients aged 18–65.

**Discussion:**

Despite the limitations of the FAERS database, these findings emphasize the importance of monitoring reproductive health in male patients, particularly those of childbearing age, and highlight the need to balance ASM efficacy with potential reproductive toxicity in clinical practice. Further research is needed to validate these findings and explore underlying mechanisms.

## 1 Introduction

Antiseizure medications (ASMs) are the cornerstone of epilepsy treatment, functioning by modulating the balance between neuronal excitation and inhibition (1). Their mechanisms primarily involve enhancing gamma-aminobutyric acid (GABA)-ergic inhibitory neurotransmission or suppressing glutamatergic excitatory signaling, specifically through blocking sodium channels, amplifying GABA-mediated chloride influx, and antagonizing N-methyl-D-aspartate (NMDA) and alpha-amino-3-hydroxy-5-methyl-4-isoxazolepropionic acid (AMPA) receptors (2). Based on pharmacological properties, ASMs can be categorized into sodium channel blockers (e.g., carbamazepine), GABA enhancers (e.g., valproic acid), and glutamate receptor antagonists (e.g., gabapentin, perampanel), among others. Some drugs, such as lamotrigine, stabilize neural networks through multimodal mechanisms (3). In 2021, the global number of epilepsy patients reached 51.70 million, with an age-standardized prevalence rate of 658/10^5^ population (4). Geographically, the burden of idiopathic epilepsy varies 3- to 4-fold, with over 80% of the burden [including case numbers, prevalence, mortality, and Disability-Adjusted Life Years (DALYs)] concentrated in low- to middle-income countries (5). In terms of gender distribution, the age-standardized prevalence rate of epilepsy is slightly higher in males (322/10^5^) than in females (293/10^5^) (4). This male predominance in prevalence is supported by a comprehensive meta-analysis, which further indicates that the incidence of epilepsy may also be higher in males (6). Notably, the International League Against Epilepsy (ILAE) specifically recommends monitoring the reproductive function of patients on long-term ASMs therapy, indicating a potential link between epilepsy, its treatment, and male reproductive health (7–9).

Infertility is a global health issue defined by the World Health Organization (WHO) as the inability to achieve pregnancy after at least 12 months of regular, unprotected sexual intercourse (10). Statistically, approximately 15% of couples worldwide experience infertility, with male-factor infertility contributing to nearly 50% of these cases (11). Epidemiological data indicate that the lifetime prevalence of infertility is highest in the WHO Western Pacific Region (23.2%) and lowest in the Eastern Mediterranean Region (10.7%), while the period prevalence is highest in the African Region (16.4%) and lowest in the Eastern Mediterranean Region (10.0%) (12). In recent years, there has been growing interest in understanding the impact of epilepsy and its therapeutic medications on male reproductive health. Several case-control studies have demonstrated that in men with epilepsy, semen quality may be compromised not only due to the disease itself but also as a result of certain ASMs, which can further reduce sperm motility, increase abnormal sperm morphology, and significantly decrease semen volume and normal sperm counts (13–15). These reproductive dysfunctions may be attributed to ASMs disrupting the homeostasis of the hypothalamic-pituitary-gonadal (HPG) axis (16–19). To further investigate the potential association between ASMs and male infertility, this study conducted an in-depth analysis using the FAERS database.

In the field of drug safety evaluation, the U.S. FDA Adverse Event Reporting System (FAERS) is the largest global post-marketing drug surveillance database, integrating spontaneous reports from healthcare professionals, consumers, and pharmaceutical companies (20). It plays a pivotal role in the early detection of potential drug safety signals. The extensive scale of FAERS data enables the screening for rare or long-term adverse drug events, generating valuable hypotheses about potential associations between medications and specific health outcomes, such as reproductive dysfunction (20). Its accessibility and comprehensive coverage make it an essential tool for initial pharmacovigilance screening, particularly for evaluating complex drug-health interactions (21). It is crucial to note that FAERS data are primarily suited for hypothesis generation due to inherent limitations such as under-reporting, lack of denominator data, and confounding. This study leverages the FAERS database to conduct a disproportionality analysis for adverse events related to male infertility associated with ASMs. Our primary aim is to detect and characterize potential safety signals that warrant further investigation, rather than to establish definitive causal relationships or quantify exact risks. The findings are expected to highlight areas of concern for clinical vigilance and inform the design of more rigorous epidemiological studies to validate any signals detected and explore underlying mechanisms.

## 2 Methods

### 2.1 Data source

This study utilized data from the FDA Adverse Event Reporting System (FAERS) database, a publicly available repository containing safety reports submitted by patients, healthcare professionals, and pharmaceutical companies (available at: https://fis.fda.gov/extensions/FPD-QDE-FAERS/FPD-QDE-FAERS.html). The FAERS database adheres to the International Council for Harmonization of Technical Requirements for Pharmaceuticals for Human Use (ICH) E2B guidelines, with adverse events coded using Preferred Terms (PT) from the Medical Dictionary for Regulatory Activities (MedDRA). The database comprises seven primary files, including demographic and administrative information (DEMO), adverse event information (REAC), patient outcomes (OUTC), drug information (DRUG), therapy start and end dates (THER), report sources (RPSR), and indications for use or diagnosis (INDI). As this study used de-identified, publicly available data, it did not require institutional review board approval.

### 2.2 Data extraction and processing

**Figure 1:**
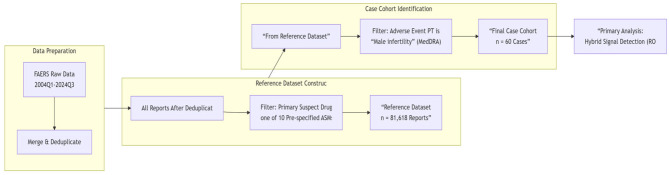
Study flowchart. This horizontal diagram illustrates the three-stage process of case selection. The reference dataset (*n* = 81,618) was created by selecting reports where one of 10 pre-specified antiseizure medications was listed as the “Primary Suspect Drug.” The final case cohort (*n* = 60) was derived by selecting reports with the Preferred Term “Male infertility” from this reference dataset. ASM, antiseizure medication; FAERS, FDA Adverse Event Reporting System; MedDRA, Medical Dictionary for Regulatory Activities; PT, Preferred Term.

Adverse events related to male infertility were identified using the MedDRA Preferred Term (PT) “male infertility.” FAERS data from January 1, 2004, to September 30, 2024, were extracted. The data extraction and case selection process is illustrated in Figure 1.

First, to construct a relevant reference dataset for disproportionality analysis, we identified all reports in which any of the following 10 pre-specified ASMs was listed as the primary suspect drug: carbamazepine, clonazepam, diazepam, lamotrigine, levetiracetam, oxcarbazepine, phenobarbital, phenytoin, topiramate, and valproic acid (and its salts). The choice of these ASMs was based on their common clinical use and previously reported associations with reproductive function. This step yielded a deduplicated reference dataset of 81,618 reports. Duplicate reports were removed by retaining the most recent FDA_DT entry for reports with the same CASEID; if CASEID and FDA_DT were identical, the report with the higher PRIMARYID was retained.

Subsequently, from this reference dataset, we selected all reports where the adverse event was coded as “male infertility.” This resulted in 60 cases for the primary analysis. By defining our reference dataset based on ASMs as the primary suspect, we aimed to increase the specificity of signal detection, though confounding by concomitant medications remains a limitation. Additionally, the FAERS database lacks reliable data on the duration of drug exposure, which is a critical limitation when evaluating long-term outcomes such as infertility.

### 2.3 Data analysis

We employed a hybrid signal detection framework combining non-Bayesian (Reporting Odds Ratio, ROR) and Bayesian (Bayesian Confidence Propagation Neural Network, BCPNN) methods to detect potential reporting associations between ASMs and male infertility within the defined reference dataset. This approach leverages the complementary strengths of both methods: ROR, a frequency-based measure, was used for initial signal detection, while the Information Component (IC) of BCPNN, a Bayesian measure, is considered more robust for analyzing low-frequency data. A contingency table was constructed for each drug-adverse event pair, using the 81,618 ASM-related reports as the background. Signals were detected based on the following criteria: a positive signal required both the lower limit of the 95% confidence interval (CI) for ROR to be greater than 1 and the lower limit of the 95% CI for IC (IC_025) to be greater than 0. The term “signal” herein refers to a statistically significant disproportionate reporting relationship, not a confirmed causal risk. This dual-method integration is intended to improve the robustness of signal detection, particularly for small sample sizes.

### 2.4 Statistical analysis

Statistical significance was defined as a two-tailed *p-value* < 0.05. Data analysis and visualization were conducted using Microsoft Office 2019 and R version 4.3.3 (available at: https://posit.co/download/rstudio-desktop/). Given the small number of cases for several drugs, results are presented with emphasis on point estimates and their 95% confidence intervals, acknowledging the associated statistical instability.

### 2.5 Sensitivity analysis

To assess the robustness of the primary ROR-BCPNN framework, a sensitivity analysis was conducted using two additional disproportionality methods: the Proportional Reporting Ratio (PRR) and the Empirical Bayesian Geometric Mean (EBGM). Positive signal criteria for each method were: PRR (PRR ≥ 2, χ^2^ ≥ 4, and ≥3 cases) and EBGM (lower 95% confidence interval of EBGM > 2). The concordance of signals across all four algorithms was evaluated to inform on the stability of findings, particularly for drugs with few reports. Results of this analysis are presented in Supplementary Table S1.

## 3 Result

From the first quarter of 2004 to the third quarter of 2024, a deduplicated reference dataset of 81,618 reports was constructed for 10 pre-specified antiseizure medications (ASMs). From this dataset, 60 individual case reports of “male infertility” were identified. The distribution among specific ASMs was as follows: Valproic Acid (20 cases), Carbamazepine (19 cases), Lamotrigine (8 cases), Levetiracetam (7 cases), Oxcarbazepine (3 cases), with one case each for Phenytoin sodium, Clonazepam, and Topiramate. No cases were reported for Phenobarbital and Diazepam.

Table 1 summarizes the demographic and reporting characteristics of the broader reference dataset (*n* = 81,618), which provides context for the reporting environment of these ASMs. Within this background data, the majority of reports across all adverse events fell into the 18–65 year age group. It should be noted that this observed pattern reflects the general reporting population in FAERS and, in the absence of exposure denominators and with substantial missing data, does not support specific inferences about age-related risk for male infertility. Reports were primarily submitted by consumers (CN) and health professionals (HP/OT). Regarding reported outcomes for all events, life-threatening outcomes were noted in a considerable proportion.

**Table 1:** Demographic and reporting characteristics of the total reference dataset (*n* = 81,618).

**Characteristics **	**Oxcarbazepine (*N*, %) **	**Phenobarbital (*N*, %) **	**Phenytoin sodium (*N*, %) **	**Valproic Acid (*N*, %) **	**Diazepam (*N*, %) **	**Carbamazepine (*N*, %) **	**Lamotrigine (*N*, %) **	**Clonazepam (*N*, %) **	**Topiramate (*N*, %) **	**Levetiracetam (*N*, %) **
Total number of reports	3,648	NA	7047	14284	NA	11,178	13,817	7,063	5,193	19,388
**Gender**										
Male		3,648 (100%)	7,047 (100%)	14,284 (100%)		11,178 (100%)	13,817 (100%)	7,063 (100%)	5,193 (100%)	19,388 (100%)
**Weight**										
< 50 kg	303 (8.3%)		227 (3.2%)	780 (5.5%)		569 (5.1%)	1,126 (8.1%)	135 (1.9%)	491 (9.5%)	1,482 (7.6%)
50–100 kg	596 (16.3%)		1662 (23.6%)	2154 (15.1%)		1,868 (16.7%)	1,851 (13.4%)	1,195 (16.9%)	852 (16.4%)	2,768 (14.3%)
>100 kg	109 (3.0%)		372 (5.3%)	453 (3.2%)		288 (2.6%)	314 (2.3%)	261 (3.7%)	411 (7.9%)	462 (2.4%)
Unknown	2640 (72.4%)		4786 (67.9%)	10,897 (76.3%)		8,453 (75.6%)	10,526 (76.2%)	5,472 (77.5%)	3,439 (66.2%)	14,676 (75.7%)
**Age (year)**										
< 18	738 (20.2%)		583 (8.3%)	2,020 (14.1%)		1,414 (12.6%)	2,017 (14.6%)	471 (6.7%)	866 (16.7%)	3,295 (17.0%)
18–64.9	1,294 (35.5%)		3,267 (46.4%)	6,137 (43.0%)		4,684 (41.9%)	6,485 (46.9%)	3,604 (51.0%)	2,214 (42.6%)	7,606 (39.2%)
65–85	265 (7.3%)		1,180 (16.7%)	1,286 (9.0%)		1,444 (12.9%)	992 (7.2%)	699 (9.9%)	311 (6.0%)	2,815 (14.5%)
>85	42 (1.2%)		82 (1.2%)	109 (0.8%)		119 (1.1%)	66 (0.5%)	64 (0.9%)	13 (0.3%)	372 (1.9%)
Unknown	1,309 (35.9%)		1,935 (27.5%)	4,732 (33.1%)		3,517 (31.5%)	4,257 (30.8%)	2,225 (31.5%)	1,789 (34.5%)	5,300 (27.3%)
**Reported person**										
Consumer (CN)	1,560 (42.8%)		1,906 (27.0%)	3,449 (24.1%)		3,783 (33.8%)	6,477 (46.9%)	2,949 (41.8%)	1,772 (34.1%)	4,623 (23.8%)
Health professional (HP)	346 (9.5%)		550 (7.8%)	1,917 (13.4%)		1,140 (10.2%)	1,037 (7.5%)	788 (11.2%)	431 (8.3%)	2,986 (15.4%)
Lawyer (LW)	8 (0.2%)		229 (3.2%)	324 (2.3%)		18 (0.2%)	23 (0.2%)	12 (0.2%)	35 (0.7%)	16 (0.1%)
Physician (MD)	760 (20.8%)		1,498 (21.3%)	2,929 (20.5%)		2,111 (18.9%)	3,108 (22.5%)	1,766 (25.0%)	1,493 (28.8%)	7,135 (36.8%)
Other health-professional (OT)	485 (13.3%)		1,090 (15.5%)	3,414 (23.9%)		2,419 (21.6%)	1,705 (12.3%)	692 (9.8%)	888 (17.1%)	2,339 (12.1%)
Pharmacist (PH)	268 (7.3%)		1,159 (16.4%)	1,224 (8.6%)		1,004 (9.0%)	940 (6.8%)	689 (9.8%)	363 (7.0%)	1,461 (7.5%)
Registered nurse (RN)	6 (0.2%)		5 (0.1%)	12 (0.1%)		10 (0.1%)	11 (0.1%)	2 (0.0%)	9 (0.2%)	16 (0.1%)
Unknown	215 (5.9%)		610 (8.7%)	1015 (7.1%)		693 (6.2%)	516 (3.7%)	165 (2.3%)	202 (3.9%)	812 (4.2%)
**Serious outcome**										
Congenital anomaly (CA)	15 (0.4%)		24 (0.3%)	941 (6.6%)		111 (1.0%)	565 (4.1%)	31 (0.4%)	170 (3.3%)	491 (2.5%)
Death (DE)	222 (6.1%)		439 (6.2%)	1,015 (7.1%)		777 (7.0%)	892 (6.5%)	1,185 (16.8%)	269 (5.2%)	1,284 (6.6%)
Disability (DS)	61 (1.7%)		95 (1.3%)	414 (2.9%)		173 (1.5%)	278 (2.0%)	137 (1.9%)	132 (2.5%)	244 (1.3%)
Life-threatening (LT)	845 (23.2%)		2,214 (31.4%)	4,466 (31.3%)		3,415 (30.6%)	3,322 (24.0%)	1,501 (21.3%)	1,169 (22.5%)	5,384 (27.8%)
Hospitalization (HO)	156 (4.3%)		372 (5.3%)	705 (4.9%)		565 (5.1%)	778 (5.6%)	345 (4.9%)	237 (4.6%)	928 (4.8%)
Other serious outcomes (OT)	1,753 (48.1%)		2,638 (37.4%)	4,662 (32.6%)		4,637 (41.5%)	3,967 (28.7%)	1,941 (27.5%)	1,920 (37.0%)	7,799 (40.2%)
Required intervention (RI)	12 (0.3%)		41 (0.6%)	100 (0.7%)		25 (0.2%)	46 (0.3%)	13 (0.2%)	30 (0.6%)	12 (0.1%)
Unknown	584 (16.0%)		1,224 (17.4%)	1,981 (13.9%)		1,475 (13.2%)	3,969 (28.7%)	1,910 (27.0%)	1,266 (24.4%)	3,246 (16.7%)

This table presents demographics for all ASM-related adverse event reports (not specific to infertility) in the total reference dataset.

An analysis of reporting countries for the entire reference dataset showed that the United States submitted the most reports (34,180 cases, 41.8%), followed by France (6,334 cases, 7.8%) and the United Kingdom (5,023 cases, 6.2%) (Table 2). These figures pertain to all adverse events for the selected ASMs, not specifically to infertility reports.

**Table 2:** The top five countries reporting the highest number of ASMs-related adverse events.

**Reported countries (top five)**	***N*, %**
**Oxcarbazepine**	
United states	1,588 (43.5%)
Brazil	254 (7.0%)
China	214 (5.9%)
France	160 (4.4%)
Germany	120 (3.3%)
**Phenytoin sodium**	
United states	4,257 (60.4%)
United kingdom	458 (6.5%)
India	276 (3.9%)
Canada	255 (3.6%)
Japan	215 (3.1%)
**Valproic Acid**	
United states	4,754 (33.3%)
France	2,319 (16.2%)
China	998 (7.0%)
United kingdom	711 (5.0%)
Italy	443 (3.1%)
**Carbamazepine**	
United states	3,380 (30.2%)
Japan	1,008 (9.0%)
France	719 (6.4%)
United kingdom	637 (5.7%)
China	592 (5.3%)
**Lamotrigine**	
United states	7,112 (51.5%)
United kingdom	1,055 (7.6%)
France	869 (6.3%)
Germany	827 (6.0%)
Japan	812 (5.9%)
**Clonazepam**	
United states	3,536 (50.1%)
France	708 (10.0%)
Canada	596 (8.4%)
Italy	388 (5.5%)
Brazil	317 (4.5%)
**Topiramate**	
United states	2,531 (48.7%)
United kingdom	482 (9.3%)
Italy	242 (4.7%)
Germany	234 (4.5%)
France	228 (4.4%)
**Levetiracetam**	
United states	7,022 (36.2%)
Germany	1,785 (9.2%)
Japan	1,676 (8.6%)
United kingdom	1,571 (8.1%)
France	1,266 (6.5%)

This ranking is based on the total number of reports for all ASM-related adverse events in the total reference dataset (n = 81,618), not specific to male infertility.

**Figure 2:**
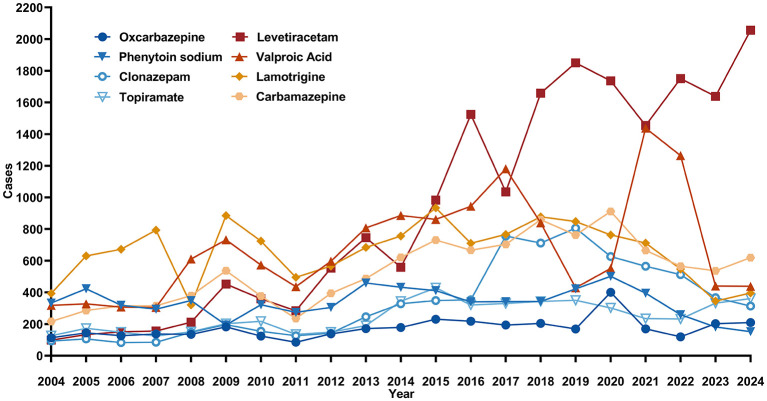
The annual distribution of AE reports related to ASMs from 2004 to 2024. Levetiracetam exhibited the most significant upward trend, increasing from 98 cases in 2004 to 2,056 cases in 2024. Other drugs, such as Phenytoin sodium, Valproic Acid, Clonazepam, and Carbamazepine, showed larger fluctuations with an initial increase followed by a decline. Lamotrigine reports slightly decreased, while Oxcarbazepine and Topiramate reports showed a slight increase, remaining relatively stable overall.

The annual reporting trends for all adverse events related to the eight ASMs with at least one case of male infertility are shown in Figure 2. Levetiracetam exhibited the most pronounced increase in reporting volume over time, a trend likely reflecting its growing clinical use and overall reporting frequency.

Overall, reports of male infertility constituted a very small proportion (0.07%) of all ASM-related reports in the analyzed dataset.

### 3.3 Disproportionality analysis

**Figure 3:**
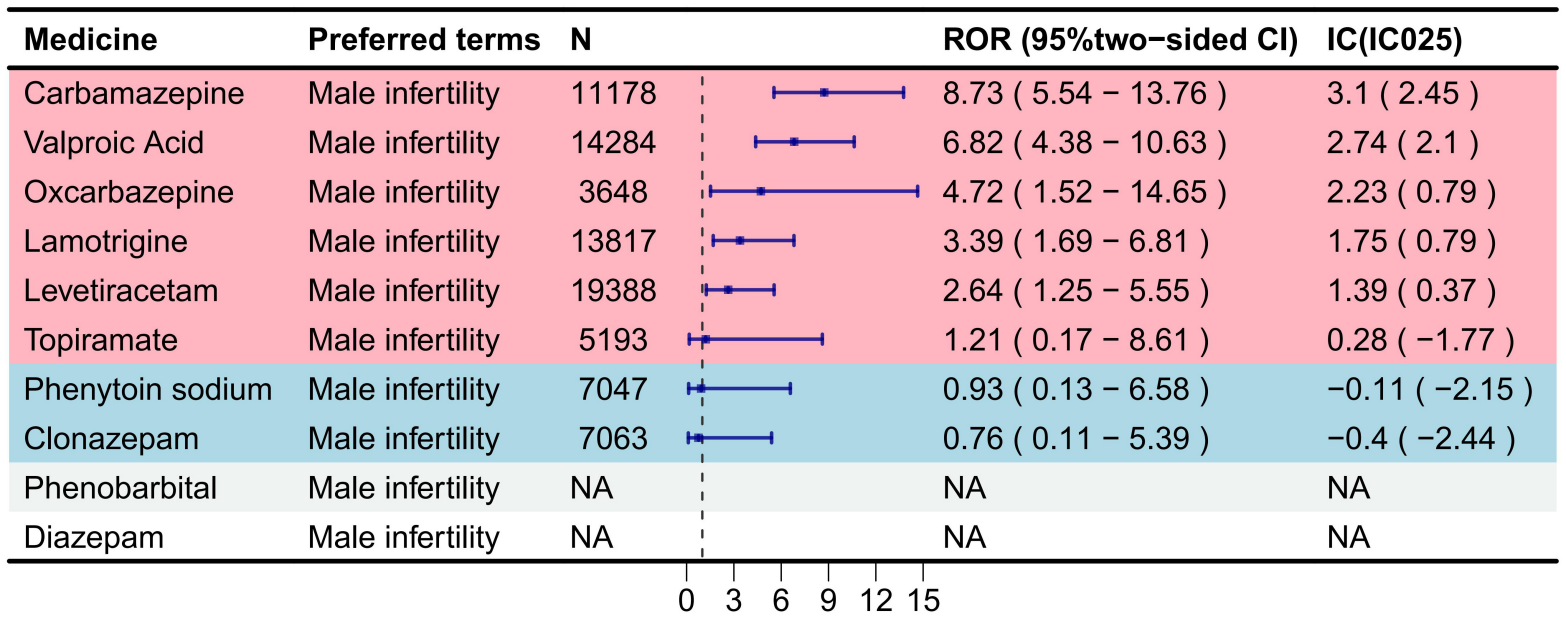
Disproportionality analysis for ASMs and male infertility (Preferred term: Male infertility). The forest plot displays the association between various antiepileptic drugs and male infertility reports in the FAERS database, measured by the Reporting Odds Ratio (ROR) with 95% confidence intervals (CI). Drugs are sorted in descending order of ROR value. The dashed vertical line represents the null value (ROR = 1). Points to the right of the line indicate a positive association, while points to the left suggest a negative or no association. A signal was considered positive if the lower limit of the 95% CI for ROR exceeded 1. Carbamazepine and valproic acid showed the strongest positive signals.

Signal detection results for antiseizure medications (ASMs) and “Male infertility” are presented in Figure 3. The forest plot ranks drugs by their Reporting Odds Ratio (ROR) point estimates. Based on pre-specified criteria (ROR 95% CI lower limit >1 and IC025 >0), five ASMs—carbamazepine, valproic acid, oxcarbazepine, lamotrigine, and levetiracetam—demonstrated positive signals from both the ROR and Bayesian Confidence Propagation Neural Network (BCPNN) methods, indicating statistically significant disproportionate reporting associations.

Carbamazepine exhibited the strongest association (ROR = 8.73, 95% CI: 5.54–13.76; IC = 3.10, IC025 = 2.45), followed by valproic acid (ROR = 6.82, 95% CI: 4.38–10.63; IC = 2.74, IC025 = 2.10), oxcarbazepine (ROR = 4.72, 95% CI: 1.52–14.65; IC = 2.23, IC025 = 0.79), lamotrigine (ROR = 3.39, 95% CI: 1.69–6.81; IC = 1.75, IC025 = 0.79), and levetiracetam (ROR = 2.64, 95% CI: 1.25–5.55; IC = 1.39, IC025 = 0.37). The elevated point estimates suggest a higher reporting likelihood for these drugs. Of note, the positive signals for oxcarbazepine, lamotrigine, and levetiracetam were derived from a limited number of cases (*n* = 3, 8, and 7), with the wide confidence intervals reflecting the associated statistical uncertainty.

Conversely, phenytoin sodium, topiramate, and clonazepam did not meet the positive signal criteria in either analysis. In summary, these findings reveal a disproportionate reporting association between specific ASMs and male infertility within the FAERS database.

## 4 Discussion

To better contextualize the clinical significance of our findings, it is important to consider the baseline prevalence of infertility in the general population. Infertility affects approximately 10%−15% of couples attempting to conceive, with a male factor contributing solely or partially in about one-quarter of these cases (22). Furthermore, the global burden of male infertility is substantial and has been steadily increasing. A study on the global burden of disease revealed that the age-standardized prevalence rate of male infertility rose by 19% from 1990 to 2019, with the peak burden observed in the 30–34 age group—the primary demographic for both family planning and ASMs therapy (23). Against this background, the identification of 60 cases of male infertility in the FAERS database, while a numerically small subset, represents a potentially significant signal. This is particularly true given the substantial underreporting inherent in spontaneous reporting systems and the fact that these cases occurred in a population where a prescribed medication is a suspected contributor. The key question is not merely the absolute number of reports, but the strength of the disproportionality signal and the potential for ASMs to add an incremental risk on top of the baseline population rate of male infertility. This perspective strengthens the argument for heightened clinical vigilance.

In terms of drug reporting proportions, the usage trends of second-generation ASMs have undergone significant changes, while the reporting rates of first-generation ASMs have shown a declining trend, reflecting the substitution effect of newer drugs in clinical practice. Newer ASMs, such as levetiracetam (LEV), have gained widespread use due to their broad-spectrum antiepileptic properties, favorable pharmacokinetic profiles, and lower risks of drug interactions (13, 24, 25). Consequently, the number of adverse event reports for LEV has surged from fewer than 100 cases in 2004 to over 2,000 cases in 2024. In contrast, the reporting rates for first-generation ASMs such as phenytoin and valproic acid have been decreasing year by year. The reporting rate for lamotrigine (LTG) has remained relatively stable, while those for oxcarbazepine (OXC) and topiramate (TPM) have shown a slight increase, likely due to their growing use in specific epilepsy syndromes. The substitution effect of newer ASMs is primarily reflected in the following aspects: first, compared to first-generation ASMs, newer ASMs generally have lower risks of drug interactions and demonstrate better tolerability in older adult patients, thereby reducing adverse events in clinical use (26). Second, certain newer ASMs, such as lamotrigine, are not only used for epilepsy but also widely applied in other neurological and psychiatric disorders, showcasing broader indications and clinical potential (27). Additionally, early studies suggest that first-generation ASMs like phenobarbital, phenytoin, and valproic acid may adversely affect cognitive function, whereas newer ASMs exhibit advantages in this regard, further reducing the occurrence of related adverse events (28). Despite the progress made by newer ASMs in replacing first-generation ASMs, the latter remain important treatment options in certain scenarios due to their efficacy and cost-effectiveness (29).

This study employed disproportionality analysis to quantify the risk of male infertility associated with different ASMs. The results indicated that carbamazepine (CBZ) and valproate (VPA) posed the highest risks, potentially by disrupting the hypothalamic-pituitary-gonadal (HPG) axis, impairing testosterone synthesis, inducing mitochondrial dysfunction, and directly affecting testicular tissue, leading to reproductive dysfunctions such as oligospermia and azoospermia (15, 30). These findings are consistent with the meta-analysis by Asghar et al. (14) and the animal experiments by Sukhorum and Iamsaard (31), which further confirmed that VPA impairs testicular function and male fertility through multi-target mechanisms. Specifically, VPA was shown to reduce the expression of Ki-67, cholesterol side-chain cleavage enzyme (P450scc), and phosphorylated proteins (41, 51, and 83 kDa), while increasing the expression of steroidogenic acute regulatory protein, thereby interfering with testosterone synthesis and spermatogenesis (31). Histopathological analysis further demonstrated VPA's direct damage to testicular tissue, including fibrosis of the tunica albuginea and tubular basement membranes, as well as an increase in premature acrosome reactions. However, human data remain limited, with some clinical observations indicating that long-term VPA use in male patients is often associated with reduced sperm concentration, motility, and morphology, though the specific mechanisms require further validation (25). In contrast, oxcarbazepine (OXC), lamotrigine (LTG), and levetiracetam (LEV) were associated with lower risks of male infertility. Multiple case-control studies have shown that OXC significantly improves sperm motility and survival rates, while LTG and LEV have minimal effects on sexual function and hormone levels, likely due to their limited impact on the HPG axis and testosterone levels (32). A prospective study by Markoula et al. (33) also supports that switching from VPA to LTG or LEV can improve sperm parameters and increase the chances of natural conception. However, clinical research by Ceylan et al. (34) suggests that LEV may reduce sperm parameters without altering hormone levels, while cell experiments by Wang et al. (35) indicate that VPA promotes the differentiation of human pluripotent stem cells into spermatogonial stem cells. These conflicting results highlight the need for further research to elucidate the mechanisms by which ASMs affect male reproductive function, particularly their specific targets in testicular tissue, spermatogenesis, and mitochondrial function. Additionally, phenytoin, topiramate (TPM), and clonazepam showed negative signals in both algorithms, suggesting they may either reduce the risk of male infertility or have no significant effect. Animal experiments by Abbas Jafari et al. further support the protective effects of TPM against testicular ischemia-reperfusion injury, indicating that some ASMs may have protective effects on testicular tissue (36). The interaction between ASMs and male infertility is complex and involves multiple targets and mechanisms. While some ASMs may affect male fertility by disrupting the HPG axis, inducing oxidative stress, or exerting direct toxic effects on testicular tissue (e.g., impairing Sertoli cell function), their specific targets and mechanisms require further investigation (37, 38). For example, potential targets such as GABA receptors and ion channels, may play important roles in ASM-induced male infertility (39, 40). Future research should further explore the multi-target mechanisms of ASMs on the male reproductive system, particularly their effects on testicular damage, spermatogenesis, and hormonal regulation, to optimize clinical treatment strategies and reduce the risk of male infertility.

Reproductive endocrine disorders are more prevalent in male epilepsy patients than in the general population, manifesting as symptoms such as hyposexuality, impotence, and infertility. These abnormalities may be caused by epilepsy itself or long-term use of ASMs, and are also associated with a poorer quality of life, anxiety, depression, and androgen deficiency (41**?** –43). For instance, Valproic Acid (VPA) and Carbamazepine (CBZ) not only reduce total testosterone levels but also alter the metabolism of sex hormone-binding globulin (SHBG), impacting the bioavailability of free testosterone, leading to hypogonadism, oligospermia, and sexual dysfunction (32). In contrast, Oxcarbazepine (OXC), Lamotrigine (LTG), and Levetiracetam (LEV) have a more neutral impact on sexual function and hormone levels, making them safer choices for men of reproductive age (44). For male patients planning to conceive, clinicians should prioritize ASMs with lower reproductive toxicity, such as OXC or LTG, and regularly monitor sperm parameters and hormone levels. If reproductive dysfunction occurs, switching to safer medications and supplementing with antioxidants or hormone therapy may be considered. Additionally, the widespread use of LEV underscores the need for enhanced monitoring, particularly in the primary treatment group aged 18–65. From a public health perspective, a robust pharmacovigilance system should be established to continuously monitor the safety profiles of newer ASMs, and their long-term impacts should be assessed through multicenter epidemiological investigations and cross-ethnic analyses. Meanwhile, patient education and physician training should be strengthened, and evidence-based clinical guidelines should be developed to optimize long-term management strategies for epilepsy patients, especially for protecting the reproductive health of men of childbearing age. The findings of this study provide critical evidence for clinical decision-making and public health policies, highlighting the importance of multidisciplinary collaboration and individualized treatment in reducing the reproductive toxicity of ASMs.

This study lays the groundwork for several critical future research directions. First, a more granular age analysis beyond the broad categories of < 18, 18–65, and >65 years is warranted. Given evidence that male fertility declines with advancing age (45), higher-resolution stratification (e.g., in decade increments) within the 18–65 group could clarify the interplay between ASMs exposure and age-related fertility decline. Second, prospective studies are needed to eliminate recall bias and, crucially, to systematically evaluate the contribution of female partner infertility factors, which may significantly confound the analysis if not comprehensively captured in FAERS. Finally, future research should aim to disentangle the effects of different treatment modalities on fertility, including polytherapy vs. monotherapy, and compare the reproductive impacts of ASMs against non-pharmacological interventions such as the ketogenic diet or epilepsy surgery.

This study has several limitations inherent to its design and data source. First, the voluntary nature of the FAERS database leads to under-reporting, duplicate reports, and missing information, which may affect risk quantification accuracy. Second, and critically, the number of reported male infertility cases is extremely small (*n* = 60 in total), with some drugs having only one or two reports. This makes the statistical estimates, including the Bayesian-adjusted ones, unstable and necessitates a highly cautious interpretation. The reported measures of association (e.g., ROR) should be viewed as signals of disproportionate reporting rather than precise estimates of relative risk, as FAERS lacks denominator data to calculate incidence. Third, the FAERS data lack detailed clinical context. Crucially, the analysis could not account for polytherapy regimens or the duration of drug exposure, both of which are highly relevant for an outcome like infertility. Although we considered a drug as a case if it was listed among suspect medications, the potential confounding effect of concomitant drugs cannot be ruled out. Furthermore, as a retrospective analysis, it cannot control for confounding factors such as female partner infertility and is susceptible to recall bias.

Despite these limitations, this analysis successfully generates a hypothesis and lays the groundwork for several critical future investigations. First, prospective cohort studies with detailed exposure data (duration, dosage, monotherapy/polytherapy status) are urgently needed to establish causality and control for confounders (including female factors). Second, research should stratify risks by finer age categories (e.g., by decade within the 18–65 range) given known age-related fertility decline, and compare the effects of monotherapy vs. polytherapy. Third, mechanistic studies using pharmacogenomics and molecular biology techniques are essential to unravel how ASMs like CBZ and VPA interfere with spermatogenesis, HPG axis function, and mitochondrial integrity. Finally, the long-term reproductive safety of ASMs requires continuous evaluation through integrated analyses of more robust pharmacovigilance and reproductive medicine databases.

## 5 Conclusion

This pharmacovigilance study identified a signal of disproportionate reporting between ASMs and male infertility, with the strongest signals for carbamazepine and valproate, and lower associations for oxcarbazepine, lamotrigine, and levetiracetam. Adverse event reports were most frequent in the 18–65 age group, likely reflecting higher drug exposure and reporting rates. Given the inherent limitations of spontaneous reporting databases, particularly the small number of cases and lack of exposure details, these findings should be interpreted as hypothesis-generating. They underscore the importance of considering reproductive health in the management of male epilepsy patients of childbearing age and highlight the need for further research to validate these associations, elucidate underlying mechanisms, and guide optimized, individualized treatment strategies.

## Data Availability

The original contributions presented in the study are included in the article/Supplementary material, further inquiries can be directed to the corresponding authors.
