# Supplementary material for: Analysis of ASMs and male infertility using the FDA adverse event reporting system (FAERS)
Source: Front Public Health. 2026 Jan 26;13:1738546. doi: 10.3389/fpubh.2025.1738546 (PMC12884394; doi:10.3389/fpubh.2025.1738546)
Supplement: Supplementary file 1 [file Table_1.docx]

**Supplementary Table**

Table S1. Sensitivity analysis of the association between antiseizure medications and male infertility using four disproportionality algorithms.

Supplementary Table S1 Sensitivity analysis of the association between antiseizure medications and male infertility using four disproportionality algorithms.

| Drug | Cases | Total_Reports | ROR (95% CI) | IC (IC025) | ROR | BCPNN | PRR | EBGM | Positive_Count | Concordance |
| --- | --- | --- | --- | --- | --- | --- | --- | --- | --- | --- |
| Valproic Acid | 20 | 62411 | 6.82 (4.38-10.63) | 2.74 (2.10) | ✓ | ✓ | ✓ | ✓ | 4 | All positive (4/4) |
| Carbamazepine | 19 | 46311 | 8.73 (5.54-13.76) | 3.10 (2.45) | ✓ | ✓ | ✓ | ✓ | 4 | All positive (4/4) |
| Lamotrigine | 8 | 49527 | 3.39 (1.69-6.81) | 1.75 (0.79) | ✓ | ✓ | ✓ | ✗ | 3 | Majority positive (3/4) |
| Levetiracetam | 7 | 55627 | 2.64 (1.25-5.55) | 1.39 (0.37) | ✓ | ✓ | ✓ | ✗ | 3 | Majority positive (3/4) |
| Oxcarbazepine | 3 | 13315 | 4.72 (1.52-14.65) | 2.23 (0.79) | ✓ | ✓ | ✓ | ✗ | 3 | Majority positive (3/4) |
| Topiramate | 1 | 17240 | 1.21 (0.17-8.61) | 0.28 (-1.77) | ✗ | ✗ | ✗ | ✗ | 0 | All negative (0/4) |
| Phenytoin sodium | 1 | 22541 | 0.93 (0.13-6.58) | -0.11 (-2.15) | ✗ | ✗ | ✗ | ✗ | 0 | All negative (0/4) |
| Clonazepam | 1 | 27518 | 0.76 (0.11-5.39) | -0.40 (-2.44) | ✗ | ✗ | ✗ | ✗ | 0 | All negative (0/4) |
| Phenobarbital | 0 | 791 | Not calculable | Not calculable | ✗ | ✗ | ✗ | ✗ | 0 | No cases |
| Diazepam | 0 | 23887 | Not calculable | Not calculable | ✗ | ✗ | ✗ | ✗ | 0 | No cases |

Note: Positive signals were defined by the following criteria: ROR (lower 95% CI > 1), BCPNN (IC025 > 0), PRR (PRR ≥ 2, χ² ≥ 4, and ≥3 cases), EBGM (EBGM05 > 2). ✓ indicates a positive signal; ✗ indicates no positive signal. Concordance categories: "All positive" (4/4 algorithms), "Majority positive" (3/4), "All negative" (0/4), and "No cases" (zero reports of male infertility).
